# MicroRNAs in Head and Neck Squamous Cell Carcinoma (HNSCC) and Oral Squamous Cell Carcinoma (OSCC)

**DOI:** 10.3390/cancers2020653

**Published:** 2010-04-21

**Authors:** Masashi Shiiba, Katsuhiro Uzawa, Hideki Tanzawa

**Affiliations:** Department of Clinical Molecular Biology, Graduate School of Medicine, Chiba University/1-8-1, Inohana, Chuo-ku, Chiba 260-8670, Japan; E-Mails: uzawak@faculty.chiba-u.jp (K.U.); tanzawap@faculty.chiba-u.jp (H.T.)

**Keywords:** microRNA, head and neck squamous cell carcinoma, oral squamous cell carcinoma

## Abstract

MicroRNAs (miRNAs) are small, noncoding RNAs which regulate cell differentiation, proliferation, development, cell cycle, and apoptosis. Expression profiling of miRNAs has been performed and the data show that some miRNAs are upregulated or downregulated in cancer. Several studies suggest that the expression profiles of miRNAs are associated with clinical outcomes. However, the set of miRNAs with altered expressing differs depending on the type of cancer, suggesting that it is important to understand which miRNAs are related to which cancers. Therefore, this review aimed to discuss potentially crucial miRNAs in head and neck squamous cell carcinoma (HNSCC) and oral squamous cell carcinoma (OSCC).

## 1. Introduction

Intensive investigations with innovative technologies have led to the identification of biomarkers that are useful in characterizing cancers. A class of small noncoding RNAs termed microRNAs (miRNAs) has recently been recently highlighted as the biomarker for some types of cancers. miRNAs are endogenous, small, noncoding RNAs of 17–25 nucleotides that are thought to regulate approximately 30% of human genes [1,2,3]. miRNAs were first discovered in worms, and later studies clarified that they are widely conserved in various animals and plants [3,4,5]. They are involved in essential biological activities such as such as cellular differentiation [6,7], proliferation [8,9,10], development [11,12], apoptosis [13,14,15], and regulation of cell cycle [16,17,18]. They perform these actions by regulating target gene expression through imperfect base pairing with the 3'-untranslated region (3'-UTR) of target mRNAs of protein-coding genes, leading to the cleavage of homologous mRNA or translational inhibition [1,6,7,19]. They are also differentially expressed in various types of cancers, including oral cancer, compared with noncancerous tissues, suggesting that they may have crucial roles in tumorigenesis [20,21,22,23,24]. It has been shown that there are two types of cancer-related miRNAs; oncogenic or tumor suppressor miRNA. MiR-15a, miR-16-1 are tumor suppressor miRNA targeting 3'-UTR of anti-apoptotic protein Bcl2 [25]. And let-7 family is also known as tumor suppressor miRNA that functions through inhibiting famous oncogenic mRNAs, such as *Ras*, *c-myc*, *high mobility group A2* (*HMGA2*) [26,27,28]. On the contrary, miR-155, miR-17–92 cluster, miR-21, miR-372, miR-373 are found to be oncogenic miRNAs with supporting experimental data [24]. Studies relating the expression profiling of miRNAs to diagnosis and prognosis of cancers have recently been published—some have been shown closely associated with clinical outcome [3,24]. This review aims to discuss miRNA activities in HNSCC/OSCC and clarify the roles of miRNAs as oncogenes or tumor suppressors.

## 2. miRNA Expression Profiling in HNSCC/OSCC

Most miRNA expression profiling has been performed with the microarray analysis method. Using this method, altered miRNA expression in HNSCC/OSCC has been investigated by several groups [29]. Table 1 summarizes miRNA expression profiles that have been reported recently. Some miRNAs show consistently altered expressions in different studies. For example, upregulated expression of miR-21, -31, -18, and -221 has been reported by at least two independent studies (Table 1).

Upregulated expression of miRNA-21 in tumor samples or cell lines of HNSCC/OSCC has been reported by several investigators [30,31,32,33,34,35]. MiR-21 is also upregulated in acute myeloid leukemia (AML) and chronic lymphocytic leukemia (CLL) [36,37], solid tumors of the breast, colon, pancreas, lung, prostate, liver and stomach, and glioblastoma [38,39,40,41]. These facts strongly suggest that miR-21 may be one of the most important miRNAs in various types of cancers including HNSCC/OSCC. 

Upregulated expression of miR-31 has been reported in OSCC cell lines [22,42] and in head and neck cancer cell lines [35], and there have been no reports suggesting downregulated expression of miR-31 in OSCC. While miR-31 appears to be upregulated in hepatocellular carcinoma [43] and colorectal carcinoma [44], its downregulated expression has been reported in gastric carcinoma [45] and prostate carcinoma [46]. The function of miR-31 in tumorigenesis remains unclear; the marker might have diverse biological activities depending on the type of cancer.

MiR-221 is overexpressed in head and neck cancer cell lines [35] and in HNSCC samples [34]. Furthermore, upregulation of miRNA-18 was observed in head and neck cancer cell lines [35] and in HNSCC samples and cell lines [31]. Avissar *et al*. showed upregulated expression of miR-21, -18a, and -221 and downregulated expression of miR-375, and demonstrated that an expression ratio of miR-221: miR-375 showed a high sensitivity and specificity for disease prediction in HNSCC [34]. 

**Table 1:** Expression profiling of miRNAs in HNSCC/OSCC.

Authors (year)	Altered expression of miRNAs		Materials/methods for miRNA expression profiling	Ref.
	Up	Down		
Avissar *et al*.	miR-21	miR-375	HNSCC samples/	[34]
(2009)	miR-18a		Microarray	
	miR-221		Quantitative RT-PCR	
Cervigne *et al*.	miR-21		OSCC and leukoplakia samples/	[33]
(2009)	miR-181b		TaqMan low density arrays (TLDA)	
	miR-345		Quantitative RT-PCR	
Chang *et al*.	miR-211		OSCC samples/	[63]
(2008)			TaqMan MicroRNA Assay kit	
Chang *et al*.	miR-21	miR-494	HNSCC samples and cell lines (JHU-011, JHU-012, FaDu, JHU-09)/	[31]
(2008)	let-7		Microarray	
	miR-18		Quantitative RT-PCR	
	miR-29c			
	miR-142-3p			
	miR-155			
	miR-146b			
Childs *et al*.	miR-21	miR-1	HNSCC samples/	[32]
(2009)		miR-133a	Microarray	
		miR-205	Quantitative RT-PCR	
		let-7d		
Henson *et al*.		miR-125b	OSCC samples and cell lines (UPCI: SCC084, SCC078, SCC131, SCC040, SCC029, SCC032, SCC104, SCC142, SCC116, SCC066)/Quantitative PCR	[21]
(2009)		miR-100		
Jiang *et al*.	miR-205		HNSCC cell lines (SCC17A, SCC17B, SCCD12, SCC10B, SCC5)/	[70]
(2005)			Real-time quantitative PCR	
			Northern blotting	
Kozaki *et al*.	miR-374	miR-27a	OSCC cell lines (Ca9-22, HO-1-N-1, HOC313, HOC815, HSC-2, HSC-3, HSC-4, HSC-5, HSC-6, HSC-7, KON, KOSC-2, NA, OM1, OM2, SKN3, TSU, ZA)/Real-time RT-PCR	[22]
(2008)	miR-340	miR-34b		
	miR-224	miR-34c		
	miR-10a	miR-203		
	miR-140	miR-302c		
	miR-181a	miR-23a		
	miR-146a	miR-27b		
	miR-126	miR-34a		
	miR-31	miR-215		
	miR-9	miR-299		
	miR-9*	miR-330		
		miR-337		
		miR-107		
		miR-133b		
		miR-138		
		miR-139		
		miR-223		
		miR-204		
		miR-370		
		let-7d		
		miR-95		
		miR-302a		
		miR-367		
		let-7g		
		miR-23b		
		miR-128a		
		miR-148a		
		miR-155		
		miR-200c		
		miR-302b		
		miR-368		
		miR-122a		
		miR-371		
		let-7a		
		miR-26b		
		miR-30e-5p		
		miR-96		
		miR-125a		
		miR-132		
		miR-200b		
		miR-199b		
		miR-296		
		miR-373		
		miR-137		
		miR-197		
		miR-193a		
		let-7e		
		miR-30d		
		miR-331		
		miR-342		
		miR-338		
		miR-199a		
		miR-372		
		miR-184		
Li *et al*.	miR-21		Tongue squamous cell carcinoma samples/	[30]
(2009)			Microarray	
			Quantitative reverse transcription-PCR	
			Northern blotting	
Park *et al*.		miR-125a	Saliva of oral squamous cell carcinoma patients/	[65]
(2009)		miR-200a	Reverse transcriptase-preamplification-quantitative PCR	
Tran *et al*.	miR-21	miR-200c	Head and neck cancer cell lines (FaduD, HN6, HN13, UM-SCC9, UM-SCC47, UM-SCC10A, UM-SCC11A, TUM-SCC38, UMSCC4)/MicroarrayNorthern blotting	[35]
(2007)	miR-200a	miR-373		
	miR-103	miR-345		
	miR-19a	miR-382		
	miR-361	miR-342		
	miR-27a	miR-133b		
	miR-US33-1	miR-373*		
	miR-7b	miR-US25-2-5p		
	miR-100	miR-346		
	miR-125b	miR-212		
	miR-28	miR-375		
	miR-18	miR-328		
	miR-22	miR-127		
	miR-15a	miR-154		
	miR-30b	miR-133a		
	miR-320	miR-371		
	miR-98	miR-302d		
	miR-15b	miR-302c		
	miR-200b	miR-302b		
	miR-16	miR-449		
	let-7f	miR-340		
	miR-29a	miR-378		
	let-7a			
	miR-221			
	let-7d			
	miR-23a			
	miR-31			
	miR-107			
	let-7c			
	miR-29b			
	miR-24			
	miR-23b			
	miR-205			
Wong *et al*.	miR-184	miR-133a	Tongue squamous cell carcinoma samples/Quantitative reverse transcription-PCR of mature miRNAs	[42]
(2008)	miR-34c	miR-99a		
	miR-137	miR-194		
	miR-372	miR-133b		
	miR-124a	miR-219		
	miR-21	miR-100		
	miR-124b	miR-125b		
	miR-31	miR-26b		
	miR-128a	miR-138		
	miR-34b	miR-149		
	miR-154	miR-195		
	miR-197	miR-107		
	miR-132	miR-139		
	miR-147			
	miR-325			
	miR-181c			
	miR-198			
	miR-155			
	miR-30a-3p			
	miR-338			
	miR-17-5p			
	miR-104			
	miR-134			
	miR-213			
Yu *et al*.	miR-21	miR-16	The animal model of oral squamous cell carcinoma (hamster)/Microarray	[62]
(2009)	miR-200b	miR-26a		
	miR-221	miR-29		
	miR-338	miR-124a		
	miR-762	miR-125b		
		miR-126-5p		
		miR-143		
		miR-145		
		miR-148b		
		miR-155		
		miR-199a		
		miR-203		

In contrast, some miRNAs appear to be consistently downregulated in HNSCC/OSCC. Diminished expressions of miRNA-133a [2,7,32,35] and miRNA-133b [22,35,42,47] have been suggested in HNSCC/OSCC. Downregulation of miR-133a has been observed in bladder cancer [48], and loss of miR-133a was associated with colorectal cancer progression [49,50]. Furthermore, decreased expression of miR-133b has also been demonstrated in bladder cancer [48], lung cancer [51], and colorectal cancer [52].

Besides miR-133a, -133b, downregulated expressions of miRNA-125a, -138, -139, -200c, -26b, -302b, -302c, -342, -371, -373, -375 have been reported by independent studies in HNSCC/OSCC (Table 1). These miRNAs show consistently altered expression in the different studies, on the other hand, varying results of miRNA expression profiling data are reported in the literatures. This discrepancy may be due to variations of analysis methods or materials. To interpret the microarray data of miRNAs precisely, Tang *et al*. have developed a highly sensitive miRNA array platform and introduced a new approach of data normalization based on Northern blot analysis [53]. Yin *et al*. described the importance of validation using a set of standard methods, such as real-time PCR analysis, for effective analysis [54]. These studies strongly suggest that we should conclude miRNA expression profiling throughout plural experimental approaches.

## 3. Functional Analysis of miRNAs in HNSCC/OSCC Cells

Studies have shown that miR-21 suppresses apoptosis [55], that inhibition of miR-21 reduces cell growth [38], and that miR-21 regulates tumor suppressor genes such as LRRFIP1 (leucine rich repeat (in FLII) interacting protein 1) [56], PTEN (phosphatase and tensin homolog) [39], PDCD4 (programmed cell death 4) [57], and TPM1 (tropomyosin 1) [58]. Thus, miR-21 is believed to be one of the oncogenic miRNAs. Liu *et al*. reported that inhibition of miR-21 with antisense oligonucleotide (ASO) reduced survival and anchorage-independent growth, and induced apoptosis in tongue squamous cell carcinoma cell lines, and that repeated injection of miR-21 ASO suppressed tumor formation in nude mice by reducing cell proliferation and inducing apoptosis. In addition, among patients with tongue SCC, those that overexpressed miR-21 showed poorer prognosis than those who did not. Chang *et al*. also reported that transfection with miR-21 inhibitor induced a statistically significant increase in cytochrome c release and increased apoptosis in HNSCC cell lines [31]. These results suggest that an inhibitory effect on cancer cell apoptosis in HNSCC/OSCC might be one of the crucial functions of miR-21.

Wong *et al*. demonstrated that tongue SCC cell lines transfected with miR-133a and miR-133b precursors displayed reduced proliferation rate and increased numbers of apoptotic cells. They also demonstrated that both miR-133a and miR-133b target transcription of pyruvate kinase type M2 (PKM2), a potential oncogene in solid cancers, suggesting that PKM2 and miR-133a and miR-133b might be associated with the tumorigenesis of tongue squamous cell carcinoma [47].

Some studies have implied that miRNAs modulate chemosensitivity or radiosensitivity. Boo *et al*. reported a correlation between increased HMGA2 expression and enhanced chemosensitivity towards doxorubicin in breast cancer cells [59]. In addition, Hebert *et al*. showed that transfection of pre-miRNA-98 decreased the expression of HMGA2 and also potentiated resistance to doxorubicin and cisplatin in HNSCC cell lines [60]. These data suggest miR-98 might be one of the factors regulating chemosensitivity in HNSCC/OSCC.

We previously identified 25 genes, which were more highly expressed in radioresistant OSCC cells than in radiosensitive cells in a dose-dependent manner [61]. Henson *et al*. demonstrated that, among these genes, ID1, MMP13, and FGFR3 were downregulated in OSCC cells transfected with miR-100 [21]. They also suggested that identification of these genes should help to elucidate the molecular mechanisms of OSCC radioresistance.

These investigations strongly suggest that certain miRNAs play crucial roles in chemo- and radiosensitivity in HNSCC/OSCC and could be biomarkers for choosing the appropriate therapies in the clinical setting (Table 2).

**Table 2:** Function of miRNAs in HNSCC/OSCC cells reported in the recent literature.

Authors (Year)	Results of functional analysis of miRNAs in HNSCC /OSCC cells	Ref.
Hebert *et al*. (2007)	Expression of high mobility group A2 (HMGA2), which is associated with enhanced selective chemosensitivity towards the topoisomerase (topo) II inhibitor, doxorubicin, was regulated in part by miR-98.	[60]
Henson *et al*. (2009)	Transfection of miR-125b and miR-100 reduced cell proliferation and modified the expression of target and nontarget genes. Some of the genes are overexpressed in radioresistant OSCC cells.	[21]
Kozaki *et al*. (2008)	Transfection of miR-137 or miR-193a reduced cell growth, with down-regulation of the translation of cyclin-dependent kinase 6 or E2F transcription factor, respectively.	[22]
Liu *et al*. (2009)	Transfection of miR-138 suppressed cell invasion and led to cell cycle arrest and apoptosis. Knockdown of miR-138 enhanced cell invasion and suppressed apoptosis.	[71]
Liu *et al*. (2009)	Transfection of miR-222 reduced the expression of matrix metalloproteinase 1 (MMP1) and manganese superoxide dismutase 2 (SOD2). The data indicated that miR-222 inhibits invasion of oral tongue squamous cell carcinoma.	[72]
Chang *et al*. (2008)	Transfection of miR-21 increased cell growth, and transfection of the miR-21 inhibitor caused decreased cell growth. Flow cytometry analysis showed that mir-21 inhibitor transfection induced signiﬁcant increase in cytochrome c release and increased apoptosis.	[31]
Chang *et al*. (2008)	Enforced miR-211 expression increased proliferation, migration, and anchorage-independent colony formation, suggesting that high *miR-211* expression may be associated with the progression of oral carcinoma.	[63]
Li *et al*. (2009)	Inhibiting miR-21 with antisense oligonucleotide (ASO) reduced survival and anchorage-independent growth, and induced apoptosis. Repeated injection of miR-21 ASO suppresses tumor formation in nude mice by reducing cell proliferation and inducing apoptosis	[30]
Wong *et al*. (2008)	Transfection of miR-133a and miR-133b precursors reduced Pyruvate Kinase type M2 (PKM2) expression was reduced.	[47]
Wong *et al.* (2008)	Inhibition of miR-184 reduced cell proliferation rate and induced down-regulation of c-Myc. Suppressing miR-184 induced apoptosis.	[42]

## 4. Animal Models

Yu *et al*. established an animal model of OSCC to investigate expression profiles of miRNAs in oral carcinogenesis [62]. Expressions of miRNAs were compared between the experimental group animals, which received a carcinogen, and the control animals, which did not. They identified five upregulated miRNAs (hsa-miR-21, hsa-miR-200b, hsa-miR-221, hsa-miR-338, and mmu-miR-762) and twelve downregulated miRNAs (hsa-miR-16, hsa-miR-26a, hsa-miR-29a, hsa-miR-124a, hsa-miR-125b, mmu-miR-126-5p, hsa-miR-143, hsa-miR-145, hsa-miR-148b, hsa-miR-155, hsa-miR-199a, and hsa-miR-203) in cancer tissues. Some of these miRNAs have been identified as differentially expressing miRNAs in previous studies using human HNSCC/OSCC samples (Table 1). Therefore, as well as analyses of human samples or human cell lines, those using animal models could contribute to clarifying the roles of miRNAs in OSCC/HNSCC.

## 5. miRNAs as a Biomarker Associated with HNSCC/OSCC

Several investigators have emphasized the significant roles of miRNAs as biomarkers for HNSCC/OSCC. Recently reported properties of miRNAs as biomarkers in HNSCC/OSCC cells are summarized in Table 3.

An association between higher miR-211 expression and most advanced nodal metastasis, vascular invasion, and poor prognosis has been demonstrated in OSCC [63]. Furthermore, Avissar *et al*. showed that an expression ratio of upregulated miR-221and downregulated miR-375 showed a high sensitivity and specificity for disease prediction. They concluded that miR-221 and miR-375 should be evaluated as diagnostic biomarkers for prevention and treatment strategies for HNSCC [34].

Cervigne *et al*. aimed to an identify miRNA signature as a potential predictor of malignant transformation and showed that expressions of miR-21, miR-181b, and miR-345 were consistently increased and associated with lesion severity. They further suggested that these miRNAs may be potential biomarkers to assess risk of malignant transformation in oral leukoplakia [33].

Childs *et al*. examined the expression levels in primary human HNSCC tumors and found that a low level of miR-205 was significantly associated with loco-regional recurrence. Furthermore, a combination of low levels of both miR-205 and let-7d are closely related to poor survival [32]. In addition, analyses measuring the amount of specific miRNAs in samples other than the tumor lesion have been attempted to assess miRNAs as biomarkers of HNSCC/OSCC.

Originally, microarray analysis revealed seven cancer-related mRNA biomarkers (IL8, IL1B, DUSP1, HA3, OAZ1, S100P, and SAT) that exhibited at least a 3.5-fold elevation in saliva in OSCC patients, suggesting that a panel of saliva mRNAs could be used for oral cancer detection [64]. Based on this investigation, Park *et al*. measured the presence of a total of 314 miRNAs in saliva and found significantly lower salivary levels of miR-125a and miR-200a in OSCC patients than in healthy controls [65]. They conclude from this that salivary miRNAs as well as salivary mRNAs can be used as potential diagnostic markers for oral cancer.

Since overexpression of miR-184 was found in tongue squamous cell carcinoma tissues, Wong *et al*. measured plasma levels of miR-184 to determine whether the altered expression levels of miR-184 could be detected in the circulation of patients with tongue squamous cell carcinoma [42]. Plasma miR-184 levels were significantly higher in tongue squamous cell carcinoma patients when compared with normal individuals, and the levels appeared to be reduced after surgical removal of the primary tumors. Furthermore, Liu *et al*. also revealed that plasma miR-31 was significantly elevated in OSCC patients compared with age and sex-matched control individuals, and miR-31 in plasma was remarkably reduced after surgery [66]. These data indicate that both miR-184 and miR-31 might be an oncogenic miRNA in OSCC, and that its detection in plasma could a clinically useful approach.

**Table 3:** Properties of miRNAs as biomarkers in HNSCC/OSCC reported in the recent literature.

Authors (Year)	Properties of miRNAs as biomarkers	Ref.
Avissar *et al*.(2009)	The expression ratio of “miR-221: miR-375” exhibited the strongest predictive ability for differentiating HNSCC tumor from non-diseased epithelia.	[34]
Cervigne *et al*. (2009)	Expression of miR-21, miR-181b, and miR-345 was consistently upregulated and associated with increased severity during progression.	[33]
Chang *et al*. (2008)	MiR-211 expression was higher in tumors with nodal metastasis or vascular invasion than less aggressive tumors. Patients with high miR-211 expression had worse survival rates than the other groups. The data suggested that miR-211 expression could be a valuable prognostic indicator.	[63]
Childs *et al*. (2009)	Low expression levels of miR205 were associated with loco-regional recurrence. A combination of low levels of miR-205 and low levels of let-7d expression was significantly associated with poor survival rates.	[32]
Park *et al*. (2009)	Expression levels of miR-200a and miR-125a were significantly lower in the saliva of OSCC patients.	[65]
Wong *et al*. (2008)	Plasma miR-184 levels were significantly higher in tongue cancer patients, and were significantly reduced after surgical removal of the primary tumors.	[42]
Liu *et al*. (2008)	MiR-31 in plasma was significantly elevated in OSCC patients, and it was remarkably reduced after tumor resection.	[66]

## 6. Discussion

Tumorigenesis comprises various processes and many molecules are related to its complex mechanisms. Some of them have been suggested as candidates for cancer biomarker; however, conclusive biomarkers have not yet been established in HNSCC/OSCC. Lu *et al*. found extraordinary diversity in miRNA expression levels across cancers, and demonstrated that a relatively small number of miRNAs yields a lot of diagnostic information [67]. Nelson *et al*. introduced a new method for high-throughput miRNA detection, in which miRNAs can be isolated and profiled from formalin-fixed paraffin-embedded tissue [68]. These are advantages of miRNAs analysis compared with mRNA analysis, and strongly suggest that miRNA analysis is not only reliable but also promising in clinical application. Thus, if further studies concerning biological activities and intensive miRNA expression profiling are performed, miRNA analysis might emerge as a useful tool to improve outcomes in cancer therapy. Garzon *et al*. summarized a list of miRNAs associated with outcome in cancer, and among these, miR-21, miR-221, and miR-181b were also suggested to have potential as biomarkers in HNSCC/OSCC (Table 3) [24]. This means these miRNAs may have crucial roles in various types of cancer. In addition, other miRNAs might have properties specific to HNSCC/OSCC. Since miRNA is associated with regulation of numerous genes, identification of common target genes is necessary to understand the molecular mechanism of oncogenic or tumor suppressor miRNAs.

Although we focused on miRNAs in HNSCC/OSCC in this review, recent study revealed the role of miRNAs in salivary gland tumor. Zhang *et al*. found differentially expressed miRNA genes in pleomorphic adenomas of salivary gland [69]. Among the downregulated miRNAs, miR-20b, miR-144 and miR-375 were predicted to interact with the 3'-UTR of *Pleomorphic Adenoma Gene 1* (*PLGA1*) that is a proto-oncogene overexpressing in salivary gland pleomorphic adenomas. The finding suggests that miRNAs should have some roles in tumorigenesis of salivary gland tumors and might be biomarkers in various types of tumors appeared in head and neck region in addition to HNSCC/OSCC.

## 7. Conclusions

Many studies strongly suggest that miRNA should play crucial roles and could be a biomarker in HNSCC/OSCC. Despite improvements in the understanding of HNSCC/OSCC, the clinical outcome of the disease has not improved significantly. Thus, detailed investigations of miRNA, for example, concerning intercommunication among miRNAs and between miRNAs and other genes, altered protein expression induced by miRNAs, and site-specific miRNA expression profiling, are accordingly required before future clinical trials of therapeutic applications.
